# ICT Enabled Disease Diagnosis, Treatment and Management—A Holistic Cost-Effective Approach Through Data Management and Analysis in UAE and India

**DOI:** 10.3389/frai.2022.909101

**Published:** 2022-06-16

**Authors:** Manoj Kumar M V, Jagadish Patil, K. Aditya Shastry, Shiva Darshan, Nanda Kumar Bidare Sastry, Immanuel Azaad Moonesar, Shadi Atalla, Nasser Almuraqab, Ananth Rao

**Affiliations:** ^1^Department of Information Science and Engineering, Nitte Meenakshi Institute of Technology, Bangalore, India; ^2^Department of Computer Science and Engineering, Nitte Meenakshi Institute of Technology, Bangalore, India; ^3^Ramaiah Medical College and Hospitals, Bangalore, India; ^4^Mohammed Bin Rashid School of Government, Dubai, United Arab Emirates; ^5^College of Engineering and Information Technology, University of Dubai, Dubai, United Arab Emirates; ^6^Dubai Business School, University of Dubai, Dubai, United Arab Emirates

**Keywords:** SDG-3, SDG-8, virtual health clinics (VHC), Primary health centers (PHC), point of care (POC), out of pocket expenses, SDG-9

## Abstract

This concept paper addresses specific challenges identified in the UN 2030 Agenda Sustainable Development Goals (SDG) as well as the National Health Policy of India (NHP-India) and the Ministry of Health Policy of UAE (MHP-UAE). This policy calls for a digital health technology ecosystem. SDG Goal 1 and its related objectives are conceptualized which serves as the foundation for Virtual Consultations, Tele-pharmacy, Virtual Storage, and Virtual Community (VCom). SDG Goals 2 and 3 are conceptualized as Data Management & Analytical (DMA) Architecture. Individual researchers and health care professionals in India and the UAE can use DMA to uncover and harness PHC and POC data into practical insights. In addition, the DMA would provide a set of core tools for cross-network initiatives, allowing researchers and other users to compare their data with DMA data. In rural, urban, and remote populations of the UAE and India, the concept augments the PHC system with ICT-based interventions. The ICT-based interventions may improve patient health outcomes. The open and flexible design allows users to access various digital materials. Extendable data/metadata format, scalable architecture for petabyte-scale federated discovery. The modular DMA is designed using existing technology and resources. Public health functions include population health assessment, policy development, and monitoring policy implementation. PHC and POC periodically conduct syndromic surveillance to identify population risk patterns. In addition, the PHC and POC deploy medical and non-medical preventive measures to prevent disease outbreaks. To assess the impact of social and economic factors on health, epidemiologists must first understand diseases. Improved health due to compliance with holistic disease treatment plans and access to scientific health information.

## Introduction

Technological advancements/new discoveries/innovations are transforming care delivery methods and enhancing patient care experiences. It is required to develop evidence on how the innovations/advancements in healthcare can be made accessible by all in a cost-effective manner. It is also a need of the hour to deploy such technological advancements in the nearest point of care (POC) accessible by every common man. PoC is considered any location where patient care is provided (e.g., clinic, the bedside, home, or ambulance). Therefore, this conceptual paper focuses on using Patient-Centred Digital Healthcare Technologies (PC-DHT) to help provide the best health care to the local population in rural and semi-urban areas in India and remote places in UAE by setting up virtual health clinics to connect patients to the specialist doctors. After a virtual consultation and the necessary testing, the disease diagnosis is recorded in electronic health records (EHR). Once the diagnosis is complete, PC-DHT can help to draw up a detailed, holistic treatment plan. Using Web, and Mobile apps, the patient will be provided holistic treatment plans with details on the diagnosis, treatment schedule, prescription, diet, exercise etc. The data thus generated from multiple POC are made available to diverse domain researchers in the consortium of institutions through a secure and effective data management and analysis (DMA) architecture. This DMA architecture would help generate more fact-based information that aids in adaptive learning for cost-effective holistic diagnosis, treatment, and management remotely.

## Background

The current practices in healthcare delivery are blended with the help of new technological innovations. This concept paper emphasizes the design and development of the platform, which fully utilizes state-of-the-art technological advancements to improve healthcare delivery. This manuscript details the methodology for exchanging health information data and facilitating smart gadgets to capture real-time data and the methods to help the current healthcare industry. Further, this concept paper proposes an electronic approach to good health, responds to the specific challenges identified in the United Nations (UN) 2030 Agenda Sustainable Development Goals (SDG) as well as the National Health Policy of India (NHP-India) and the Ministry of Health Policy of UAE (MHP-UAE).

The uniqueness of the concept lies in the fact that the conceptual framework provides patients, including their data, access to specialist doctors from around the globe *via* the VHC; Improved health due to compliance to holistic disease treatment plans, and access to scientific health information; and Reduction in OoPE to patients.

Box 1 **Index of Acronyms.**

**Table:** 

**Index**	**Acronym Details**	**Index**	**Acronym Details**
ABS	Ayushman Bharat Scheme	NHIA	National Health Information Architecture
ADHA	Abu Dhabi Health Authority	NHIN	National Health Information Network
AI	Artificial Intelligence	NHP	National Health Policy
API	Application Programming Interface	NHP-India	National Health Policy of India
AWS	Amazon Web Services	NITI	National Institution for Transforming India
CC	Coordination Center	NLP	Natural Language Processing
CD	Communicable Diseases	NMIT	Nitte Meenakshi Institute of Technology
CDSS	Clinical Decision Support Systems	O	Objectives
DHA	Dubai health Authority	OSI	Open Systems Interconnection
DMA	Data Management & Analysis	ODSP	Open Data Solution Provider
EHR	Electronic Health Records	OoPE	Out of Pocket expenses
EIPM	Evidence-informed policy making	ORI	Offices of Research & Innovation
G	Goals	PC-DHT	Patient-Centered Digital Healthcare Technologies
GIS	Geographical Information Systems	PDA	Personal Digital Assistants
GOI	Government of India	PHC	Primary Health Centers
GPS	Geographical Position Systems	POC	Point of Care
GUI	Globally Unique Identifier	RMCH	Ramaiah Medical College & Hospitals -Bangalore-India
HP	Health professionals	SDG	Sustainable Development Goals
ICMR	Indian Council of Medical Research - New Delhi-India	SOP	Standard Operating Procedures
ICT	Information, Communication & Technology	SSP	Software Sharing Plan
IoT	Internet of Things	TP	Tele-pharmacy
IT	Information Technology	UAE	United Arab Emirates
MBRSG	Mohammed Bin Rashid School of Government -Dubai-UAE	UD	University of Dubai (UAE)
MDDS	Metadata and Data Standards	UN	United Nations
mH	Mobile Health	UN	United Nations
MHP-UAE	Ministry of Health Policy of UAE	VC	Virtual consultations
ML	Machine Learning	VCom	Virtual community
NCD	Non-communicable diseases	VHC	Virtual Health Clinics
NDHM	National Digital Health Mission	VNR	Voluntary National Review
NET	Nitte Education Trust	VPN	Virtual private network
NGO	Non-Government Organizations	VSS	Virtual storage space

### Research Problem

Today's healthcare systems in India and UAE need significant enhancement in their operation. This enhancement needs to be in the dimension of using ICT facilities in healthcare. ICT-enabled PHC will improve patient health outcomes in rural, urban, and remote populations of the UAE and India. Further, it allows patients/healthcare practitioners to access various digital materials. The focus of this concept paper is to propose a modular and scalable DMA designed based on current innovations in ICT. The creation of DMA using current advancements in ICT will facilitate Health Informatics, TeleHealth, Tele Medicine, E-Learning platforms, and Electronic Commerce.

#### Problem Description

Design and development of the platform for exchanging health information data and facilitating smart gadgets to capture real-time data are the critical aspect of the National Health Information Architecture (NHIA). The critical aspects of NHIA are in line with the Pan American Health Organization's (PAHO, 2011) Strategy and plan of action on eHealth (Scott, 2009). The goals of NHIA, PAHO, and strategy and plan of action on eHealth provide the following four critical components for facilitating cost-effective healthcare for all.

- Health Informatics: It works to combine information networks related to healthcare data. Secondly, it targets consolidating the electronic health records and related services for assimilating and analyzing the healthcare records.
- Telehealth and Telemedicine: It facilitates interaction (direct or virtual) with remote health care professionals/providers for patients with limited mobility and ill health. (National Academy of Sciences, 2015).
- E-Learning: It promotes the usage of ICTs facilities to promote education and learning opportunities to the people involved/delivering healthcare-related services. E-Learning will extend the knowledge of healthcare workers, and they can access the content at any time from anywhere. Here one can learn at their own pace and repeat the lessons. E-learning is a suitable means to educate the masses and citizens.
- Electronic commerce: Focuses on business areas of healthcare-related activities. For example: controlling services related to patients through hospital information systems (cost of the treatment, administrative information etc.).

### Objectives of the Study

To address these needs, the specific Goals and Objectives of this concept paper are:

#### Goal.1

To improve patient experiences and healthcare service delivery at the point of care, use new, patient-centered, clinician- and patient-oriented digital healthcare technology. This goal is accomplished by the following set of Objectives (O):

O.1.1 Setup Virtual Health Clinics (VHC) in Urban and Rural settings in India and remote locations in UAE over 4–5 years. VHC enables patients to consult with specialist doctors remotely to help patients get disease diagnoses and follow-ups.

O.1.2 Deploy web and mobile apps to provide health information and implement holistic disease treatment plans for patients. In addition to medication, there would be actionable instructions on diet, supplements, exercise, lifestyle modifications, and a comprehensive treatment schedule and reminders.

O.1.3 Integrate all stakeholders like doctors, nurses, and family members in designing and implementing the holistic disease treatment plans for patients using ICT.

O.1.4 Make available the electronic health record (EHR) data for disease registries, further research, and analysis while preserving the patient's privacy.

#### Goal 2

Examine how patient-centered digital healthcare technology [such as wearables, sensors, and m-Health (mH) solutions] affect patient outcomes, experiences, and the delivery of healthcare services at the point of care. The following Objectives (O) are used to achieve this goal:

O.2.1 Deploy innovative & cost-effective ICT for storing and disseminating Health Information. Integrate data from disparate sources and formats into longitudinal, standards-compliant EHR. These applications are expected to assess and evaluate the technology implementation's impact on practice workflow and quality of care.

#### Goal 3

Use advanced analytics to improve quality at the point of care. This goal is accomplished by the following set of objectives (O):

O.3.1 Test innovative digital clinical decision-making tools (such as AI and ML) that incorporate patient-generated data and patient-reported outcomes at the point of care.

O.3.2 Examine digital point-of-care systems that integrate natural language processing (NLP) with a decision support tool to transform unstructured clinical data into knowledge and make that information easier to use.

### Expected Impact and Outcomes

For the **patient**, the expected outcome from the concept is:

- *Access to specialist doctors from around the globe via the VHC*
  Rural patients are hindered by the lack of access to specialist doctors. Therefore, disease diagnosis may be delayed, and proper treatment may be difficult. The ICT-enabled virtual clinic would help patients' access specialist doctors.
- *Improved health due to compliance to holistic disease treatment plans and access to scientific health information*.
  Treatment of diseases encompasses taking the prescribed medication, adhering to the diet plan, and exercising regularly. ICT would help patients adhere to the treatment schedule and plan. The use of activity trackers would help improve the physical activity levels in patients. This would directly impact the recovery and the general health of patients. Providing adequate health information would enable patients to make better health care decisions.
- *Reduction in OoPE*
  Access to the VHC would reduce the travel expenses for disease treatment. In addition, e-prescription and e-procurement can help lower the cost of medication.

For doctors and health care workers, the expected outcome is: To have effective and efficient access to patients, including their data, beyond their geographical boundaries. Access to laboratory reports, e-prescriptions, and feedback on patient compliance is provided. The comprehensive EHR would enable doctors to make better health care decisions. The EHR would help the health care workers assess the patient's adherence to the treatment plan. The ICT would help the health care worker disseminate scientific information about diseases, their treatment, and management.

For public health policymakers and medical research, the expected outcomes are as under:

Comprehensive EHR would be beneficial in forming disease registries. The consolidated disease-specific information would spur AI/ML-based studies and provide insight into disease treatment and management by continually managing all types of data from all stakeholders, analyzing them scientifically, generating new facts, exploring patterns, and valuable for practicing healthcare professionals and patients through an adaptive learning feedback loop. This would enable the policymakers to make evidence-based health policies. The concept would have intended impact and outcomes when jointly designed and developed with different stakeholders, including the Government, PHC, Academia, and policy actors.

For the local community, the expected outcome is: Improved patients' health in the community would result in a marked improvement in the local population's health. The involvement of healthcare workers and even the family would ensure that the patient is more likely to comply with the treatment plan. The setup of VCom spurs community engagement and would improve the community's socio-economic status.

The concept would have maximum impact when the concept's goals and objectives, experiment group, and methodology are jointly discussed, designed, and developed with the local population. Adaptive learning through constant feedback, structured interviews, and focus group discussions would help derive maximum impact of the concept during implementation. Performance metrics would be designed in consultation with the local community to assess patient compliance to a comprehensive treatment plan. A dashboard with metrics to track the concept at the population level would be set up at the VHC to maximize the impact.

### The Rationale for Choosing UAE and India

According to the United Nations Development Programs (UNDP's) Human Development Index (HDI, 2020) report, the UAE is well-developed, with an HDI rating of 31, whereas India is deemed poor, with an HDI score of 131. Comparing and contrasting excellent practices in these two extreme situations allows for a more comprehensive understanding and application of the recommended principles.

### Uniqueness and Significance

Urban, Rural, and Remote Health Centers are the backbones of healthcare services in India and the UAE. The primary functions of public health include health assessment of the population, policy development, and assurance that the policies have been implemented appropriately. PHC periodically performs syndromic surveillance and attempts to identify the risk patterns in the population. The public health organization deploys can use PHC to promote medical/non-medical preventive methods to prevent disease outbreaks.

Administration of antiviral medicines, vaccination drives, medical tests etc., are some examples of clinical preventive measures. Clinical preventive measures help promote good health by preventing the spread of diseases. On the other hand, establishing quarantine facilities, providing easy access to clean drinking water, creating mass awareness of health-related information etc., are part of non-medical mitigation strategies. Epidemiologists need to understand diseases, perform statistical analysis of data, and study social and economic conditions to assess their impact on health. This is the first rationale for this proposed concept.

The second rationale stems from Voluntary National Review (VNR) 2020 report on India's and UAE's performance regarding the SDG. SDG has finetuned development policies, government priorities, businesses and citizen responsibility, and metrics for quantifying self-growth worldwide. India and UAE have implemented the SDG 2030 agenda and associated their growth priorities with the universal goal of all other countries. For the health care sector, the report identifies three challenges in India:

- Affordability and the cost of healthcare: Healthcare services offered by the public sector, while cost-effective, they are not the first choice for patients, as they are perceived as unreliable and of poor quality. The private sector is dominant in healthcare. However, there is a disparity in quality and cost of services among private health care providers;
- Health workforce density: Even though the number of midwives, nurses, and physicians per 10,000 people increased by 1.7%, it is astonishing to note that India has the lowest density of health care professionals when compared to other countries worldwide
- Lack of Health Awareness: Major gaps were identified in awareness about health care, particularly in parts like infant and adolescent health and sexual and reproductive healthcare. There is no awareness about diet and nutrition necessities, including lifestyle, mental health, and geriatric morbidity. For UAE specifically, the challenge is as mentioned under (c) above, while UAE has minimized the gaps under challenges a and b above. This concept addresses these challenges since Good Health among the population would further spur the economic growth of Indian and UAE citizens.

The third rationale is that SDG- 3 discusses India's and UAE's performance in the health care sector in terms of good health and wellbeing. India's and UAE's focus has been universally promoting preventive healthcare, empowering primary healthcare affordability, and improving the medical infrastructure. In India, the Pradhan Mantri Jan Aushadhi Pariyojana has been actively being implemented for a few years. Under Pradhan Mantri Jan Aushadhi Pariyojana, the patients are given quality medicines at effective prices. It has played an active role in decreasing patients' out-of-pocket expenses (OoPE). Additionally, the free drugs service initiative has provided the availability and accessibility of diagnostic services at the district and sub-district levels across the country—all these measures have significantly brought down OoPE.

The fourth rationale is that India and UAE are among the initial few countries to establish exact goals and pointers to reduce a load of non-communicable diseases (NCD) mortality by 25% by 2025. The growth of the National Programme for the Prevention and Control of Cancer, Diabetes, Cardiovascular Diseases, and Stroke (NPCDCS) in both nations has bolstered India's and the UAE's response to NCD. The Mental Healthcare Act was passed in 2017, and it took an entitlement approach to provide mental healthcare and services and raising awareness about mental health.

The fifth rationale is that moving toward universal health coverage, accessible, affordable, and quality health care has been institutionalized through mHealth/mH remote consultation services, and the setting up of health and wellness centers in rural, urban and remote areas has been listed as proposed health policy reforms in both UAE and India. The setting up of integrated public health labs in all districts & block level labs has also been discussed and, once implemented, would result in OoPE for the patients.

The application of ICT to COVID-19 in India and UAE is a classic case of how both countries have effectively used ICT to treat and manage the disease by exploiting the technology. The Arogya Setu App is a COVID-19 tracking mobile application produced by the Government of India. Alhosn is a comparable application in the UAE. Using cutting-edge Bluetooth technology and AI-based algorithms, both applications allow users know the danger of Corona Virus grounded on their contacts with others. The applications supplement health programs in the UAE and India to reduce COVID-19 hazards and exchange best practices. They are the fastest-growing mobile application globally, with over 100 million downloads on the major application distribution networks weeks after the launch. It currently provides 100 million customers with online medical consultations (phone and video), home lab tests, and e-pharmacy.

Another important health-related policy document is the National Health Policy (NHP), 2017. Effective use of ICT plays a vital role in achieving the goals of the NHP. For instance, the NHP 2017 recognizes the importance of digitization and wants to ensure a district-level electronic database of health information. The NHP 2017 acknowledges the key usefulness of ICT in healthcare delivery (eHealth, IoT, mHealth, and wearable electronic smart devices with customized pre-installed software suite). Similarly, NDHM works on the lines of penetrating the usage of digital health-related technologies to efficiently deliver healthcare innovations to the last person of society. Identifying the usefulness of EHR digitization, the NHP stresses the implementation of EHR electronic databases at the district level. This will further strengthen healthcare survivance and drive healthcare innovation at the level of establishing the public database/registries related to diseases of public health. It will also speed up the development of a federated learning environment for healthcare information systems, health Information Exchanges, and the National Health Information Network (NHIN) by 2025.

SDG-8 focuses on Decent Work and Economic Growth. This means that a healthy population equals a more productive workforce. Human capital investment may significantly boost a country's competitiveness. Vaccinated, healthy youngsters develop into productive workers who contribute significantly to the economy. Furthermore, healthy children free up their parents' time, allowing them to work. In Gavi-supported countries, every US dollar invested in immunization yields an additional US dollar 54 in broader social benefits such as individuals living longer and better lives (https://www.gavi.org/our-alliance/global-health-development/sustainable-development-goals).

SDG-9 emphasizes the need for Innovation, ICT, and IT Infrastructure for health care needs. The interactions between health care workers and patients would be recorded in blockchain-based systems to integrate the patient's health information into a comprehensive longitudinal record. Holistic treatment plans would be customized for the patient by health care workers. Information about the treatment plan and general information about disease management would be shared through Web and mobile apps. Cost-effective activity trackers would be provided for patients to monitor their physical activity and sleep cycles. Electronic dashboards would be set up in the PHC to assess the effect of the ICT Healthcare concept on individual patients and the local population. Involving policymakers in the early phase of research enhances the utilization of research findings in the design and delivery of national programs in both the UAE and India. The concept team is experienced in the required technical know-how.

## Study Design and Methods

This section briefs the Design/Conceptual Framework for Goal 1 (O.1.1–O.1.4), and Goals 2 & 3 and their related objectives.

### Design/Conceptual Framework for Goal 1 and Objectives O.1.1–O.1.4

How the care is given in POC can be transformed with the help of evidence-based, technology-driven solutions. These solutions may boost efficiency, minimize mistakes, improve safety, and expand patient participation and shared decision-making possibilities.

Usage of Patient-centered digital healthcare technologies (PC-DHT), Artificial Intelligence (AI) driven software/hardware, and Clinical Decision Support Systems (CDSSs) are extensively being deployed primarily for efficient and effective healthcare delivery, effective patient engagement, quality care, and to mitigating the risks of the healthcare process. For example, AI-based diagnostic treatments are currently delivered using Health AI apps (Yu et al., 2018). Many people use health-related applications installed on intelligent gadgets during day-to-day life, consisting of sensors and Patent Centered Digital Healthcare Technologies (PC-DHT), which are extensively used to deliver effective treatment outcomes (Kabelac et al., 2019).

The goal of PC-DHT is to collect the data from patients and supply the collected data to healthcare systems that can be used for informed decisions, supporting the collaborative decision-making process, and it enhances self-care quality. Healthcare professionals primarily use clinical Decision support systems to connect the healthcare knowledge at their fingertips (Baig et al., 2017). It also aids in the understanding of trends in the healthcare data and how these innovations affect the outcomes related to healthcare, delivery, and quality, which will be ultimately linked to patient experiences- this area can be explored further. Research into the utilization of novel PC-DHT systems to enhance services at the point of care correlates with the United Nations' SDG-3 goal, maximizing the value received from healthcare spending while providing 360-degree care.

With ICT and other technological advancements, the issues associated with old-aged populations (such as chronic illnesses) can be mitigated effectively. For example, people can use wearable or smart gadgets to track blood glucose, heart rate, and temperature in old age. Further, these wearable smart devices can communicate with remote devices to get diagnosed. It will help the medical fraternity constantly monitor and get alerted on the patient's conditions irrespective of the patient's location (Gadekallu et al., 2020). ICT would play a critical role in improving health care for individuals and communities worldwide (Numan et al., 2020). ICT-powered solutions would assist in closing gaps between healthcare and patients by providing new and more effective methods to access, share, and retain information, resulting in better quality treatment in less time. ICT also has the potential to improve healthcare system quality and prevent medical mistakes (Healthconnect-intl.org, 2020).

ICT is constantly adopting technological changes. It has evolved to the extent that it uses the information from various analytical and social media platforms to support the prevention of disease spread. It also encompasses the Geographical Information System to enhance its working on the problems related to epidemiology. Li et al. (2012) provided a brief description of how ICT might detect, monitor, and prevent emerging zoonotic disease outbreaks globally and in the country. This author has also highlighted how open-source and commercial technologies may be employed in pandemic prevention and human health protection activities. Shaikh et al. (2015) addressed the necessity of ICT-powered public health monitoring in dealing with increasing infectious disease threats, developing environmental and behavioral hazards, and shifting epidemiologic patterns in their study. Gomez and Katia (2010) presented ICT-driven solutions for local and global populations to promote quick response to public health catastrophes. In the case of pandemic epidemics such as HIV/AIDS, they have considered using ICT to satisfy public and health personnel's training and educational needs. They also highlighted how mobile technologies such as pagers, cell phones, Personal Digital Assistants (PDA), and tablet computers might play an essential role in crises, indicating ICT usage. Such gadgets in healthcare are referred to as mobile health (mHealth-mH). They have also stated that these mH related technologies are highly cost-effective when managing pandemics for a variety of reasons, including (a) mobile devices being reachable anywhere and at any time; (b) mobile devices being traceable through GPS; and (c) mobile devices being able to quickly obtain information (photos, video footages) and communicate in any situation.

An architecture for realizing the cloud-oriented healthcare support system has been proposed by Sandhu et al. (2016), this architecture is based on the fact and advantages of increasing usage of cloud computing technologies in day-to-day operations of information systems used to support the activities of healthcare systems. Similarly, Li et al. (2010) illustrated the method for connecting mobile-based SMS applications with eHealth and demonstrated the pandemic survivance technique in underdeveloped/developing countries. The authors have also demonstrated the method for identifying/tracing the pandemic strains. In Zhu et al. (2019) demonstrated the user-friendly PoC system that uses the advancements in IoT. The resulted data generated at PoC gets recorded through IoT and transferred to Android/iOS gadgets *via* Bluetooth/any other interfacing methods. The data can even be transferred wirelessly to the internet, which makes the data accessible immediately anywhere.

As a result, the suggested idea focuses on supplementing the PHC system with ICT-based interventions to allow cost-effective health care delivery in rural, urban, and distant populations. The ICT-based therapies can modify the health outcome and improve patients' health. The PHC is already staffed with skilled medical personnel such as nurses, midwives, and social workers. The PHC would be outfitted with generators and internet access. The infrastructure needed for a VHC would be built. A kiosk with specific cameras, monitors, microphones, and speakers would be installed in the PHC. Computers, electronic data storage, and scanners would be provided for effective data collection. A minimal diagnostic service would also be set up at the PHC. Patients would be given low-cost activity trackers, such as wearables, sensors, and mhealth/mH solutions. Patients would be taught how to utilize the activity trackers and smartphone applications. Standard operating procedures (SOP) for patient mHcare delivery will be developed and recorded in electronic form. Health care staff will be taught the use of ICT and the SOP. The EHR data would be aggregated into specialized data sets *via* disease-specific registries, but the patients' privacy and interactions would be protected. The datasets would be processed using machine learning and artificial intelligence to identify risk patterns and forecast illness outcomes and health progression. The trends discovered would aid health care professionals in refining SOP and developing evidence-based health policy.

#### The Virtual Health Clinic Infrastructure

**Figure 1:**
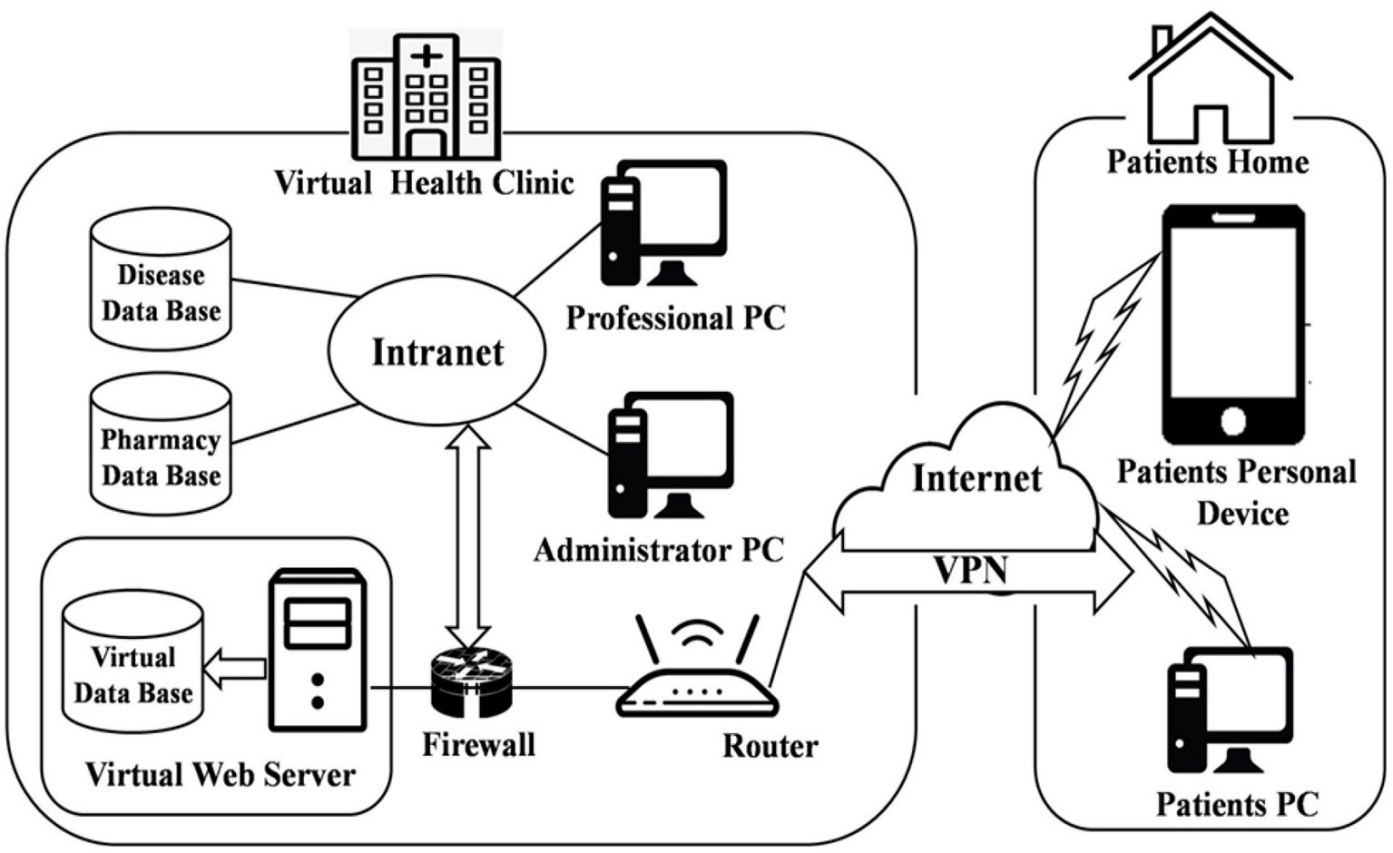
Architecture of the Virtual Health Clinic (VHC) (Source: PAHO, 2011).

Figure 1 presents the conceptual framework and approach for achieving Goal 1 and Objectives O.1.1–O.1.4 of the research concept. VHC is designed to have services like Virtual consultations (VC), Tele-pharmacy (TP), Virtual storage space (VSS), and Virtual Community (VCom). Virtual consultations would have two phases: In the first phase, appointments/consultations would be conducted *via* video-conferencing, and in the second phase, chat sessions or off-schedule consultations are considered. The EHR is made available to health professionals (HP) and patients during any of these sessions. In addition to the VC, patients may also have an in-person consultation with the HP. At the end of the appointment, the patient and the HP could set a date and time for the next session. The same approach is followed to support the Mobile assisted health care system using smartphones. The focus here is to aid the senior citizens in sticking to their medication regimes and sending reminders so that they do not skip the prescribed doses. This can also be used to inform the connected pharmacy to supply refills when low stock levels are reached.

Another service included in the VHC is tele-pharmacy (TP), which allows the pharmacist to accept electronic prescriptions and transmit drugs to the patient's house *via* courier. Patients can use TP to document their treatment history on charts and consult on available medications. The system includes a Virtual Storage Space (VSS) to hold verified information about numerous diseases, which links to other web pages for patients and HP. All links are classified according to their source and organized into categories. The virtual community (VCom) is where people may share illness information, raise awareness about various diseases, and express their thoughts or comments on articles and news items. This site is restricted to HP, where they may discuss their thoughts about their patients/cases.

VHC's architecture is divided into two stages. The first step is clinical infrastructure, in which the virtual web server is deployed to the clinic's existing demilitarized zone, which is then protected by a firewall and connected to the clinical information system network. HP connects to this server over the clinic's network. The second step is the home infrastructure, in which the patient connects to the server *via* a basic internet connection and, for security, a virtual private network (VPN).

#### Approach/Design of the Proposed Method

**Figure 2:**
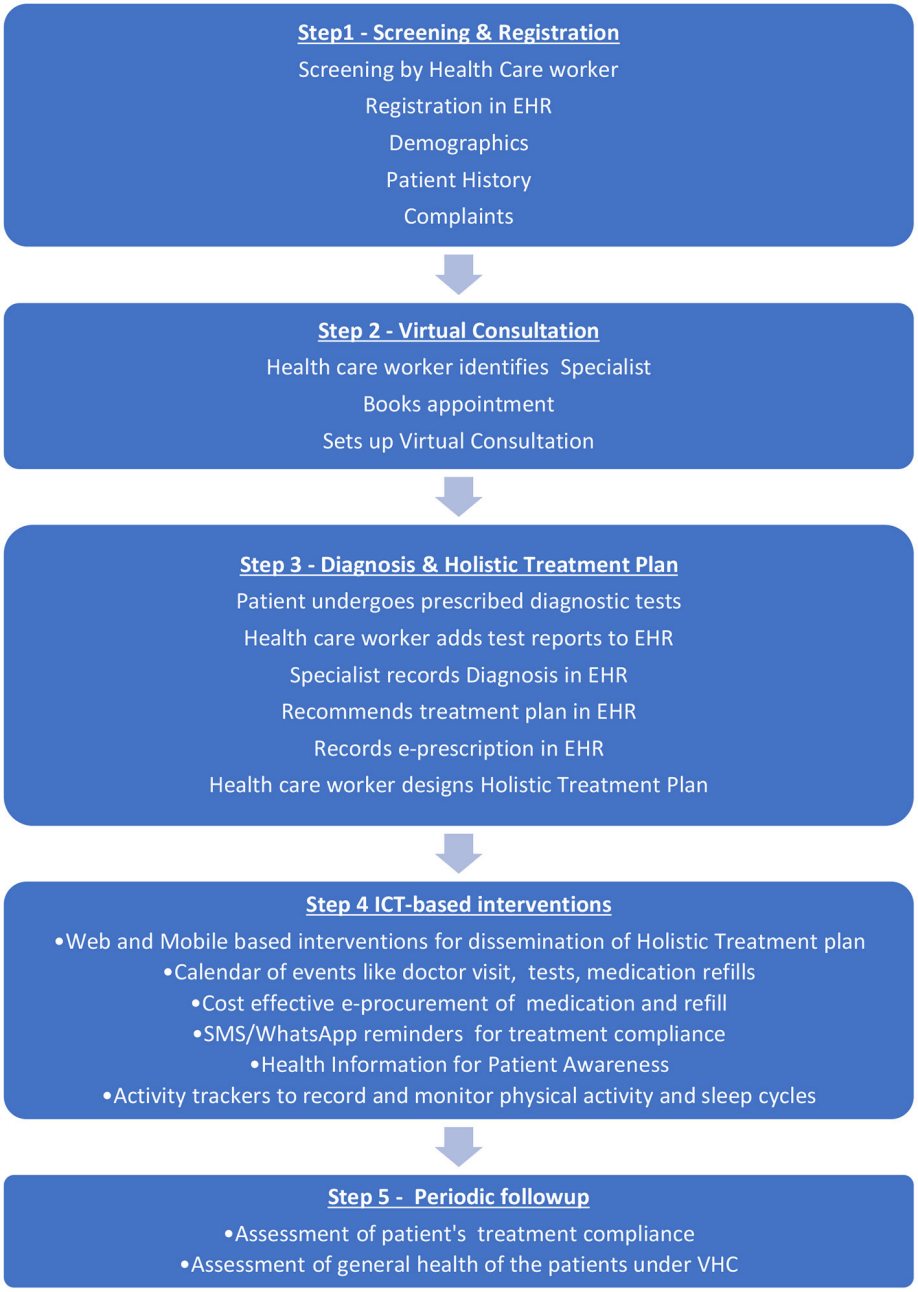
The proposed Approach for Goal 1 and O.1.1 to O.1.4 Concept (Source PAHO, 2011).

Figure 2 illustrates the proposed approach to achieve the research concept Goal 1 and O.1.1–O.1.4. The patient approaches a health care worker to register health complaints. Based on the complaints and screening, the health care worker identifies that the patient requires an expert's.

##### Consultation for a Diagnosis

The patient's consent is recorded, and the health care worker sets up a virtual consultation with a physician or specialist. The patient's history, current medical conditions, and demographics are stored in the patient's EHR. The doctor may require the patient to undergo laboratory tests, blood tests, etc. The concerned health care worker can do simple tests like blood pressure in the PHC. On completion of laboratory tests, the results are uploaded by the health care worker and made available to the consulting doctor. The reports could be text-based or even medical images. The doctor uploads the diagnosis, e-prescription, and general instructions provided for patient care. E-procurement of the medication and the refills would be done cost-effectively.

The health care worker generates a holistic treatment plan including important aspects such as medication, diet, exercise, weight management etc. An entire schedule of important dates for consultation, testing etc., would be generated for the patient. Educational material, the treatment schedule etc., are provided to the patient online, through Mobile based apps and/or social media apps such as WhatsApp. The healthcare workers would be trained to have a better health outcome. Periodically treatment compliance would be checked, and changes to the treatment schedule would be made based on patient recovery. Twice a year, the effectiveness of the mHcare initiative would be assessed to see if there is a significant improvement in patient health

### Design/Conceptual Framework for Goals 2 & 3 and Its Related Objectives

The DMA helps to turn data into valuable resources in creating business value. The tools used here can handle a high volume of data incoming through various streams at various speeds that traditional databases cannot handle. These tools create value by mining a large amount of structured, semi-structured, and unstructured data to identify patterns that can help an organization manage costs and achieve high competency efficiently. The DMA architecture also leverages existing software component technology solutions such as Machine Learning, Deep Learning, Data Mining, Data modeling, and Data Visualization. The DMA chiefly comprises the following modules: Data collection module, Data organization module, Data Analytics, Business Intelligence module, and many more. The DMA is responsible for data collection, engineering, pre-processing, analysis, and dissemination of various research endpoints.

Commoditized cloud providers, private cloud providers, or other specific configurations of commoditized hardware are used in the architecture, cloud-agnostic solutions, open-source development and public availability, and compliance with industrial and commercial standards. The architecture gives users access to cutting-edge cloud services and tools, including discounted rates on industry-leading commercial cloud environments from STRIDES Initiative partners, including Amazon Web Services (AWS) and Google Cloud. Researchers have access to consulting resources, training, billing support, and an extensive catalog of cloud services to help with data computing, storage, sharing, analysis, and sustainability. The architecture establishes the internal authority of the open-source initiative, the open-source definition, and/or Open Systems Interconnection (OSI) approved open-source licenses as a prerequisite for adopting existing internationally recognized standards or establishing the internal authority of the open-source initiative open-source definition, an OSI-approved open-source licenses.

**Figure 3:**
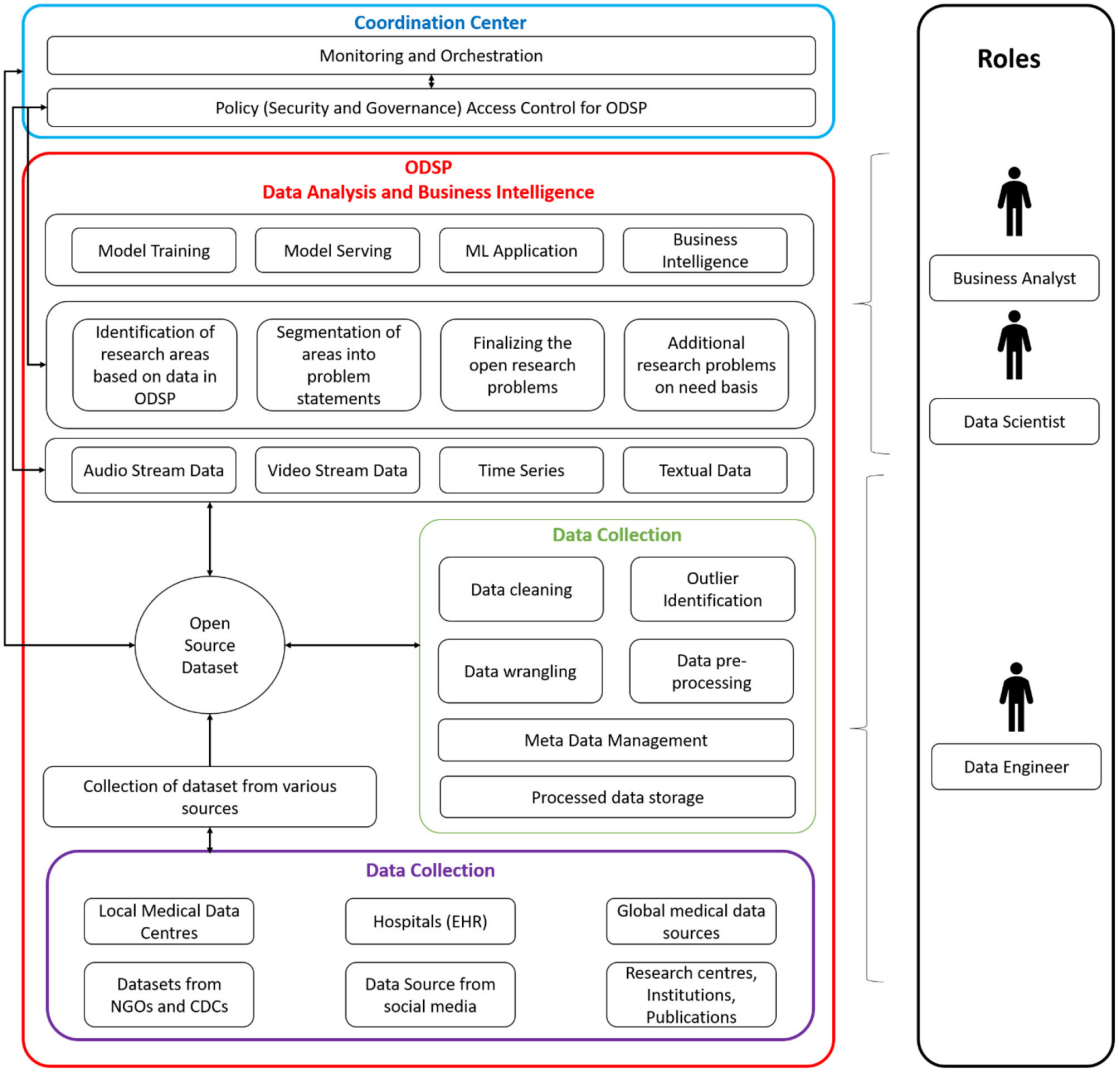
Open Architecture of Data Management for Health Sector.

The proposed DMA architecture is shown in Figure 3.

One of DMA's primary functions is data access and discovery. The suggested DMA may search and explore data inside and across scientifically relevant research to enable popular use cases. The data might be in an organized, semi-structured, or unstructured format, and it could include textual, stream, and other types of data. DMA would collaborate with the other research at partnering institutions to generate and manage data to support adopted/commonly used data standards, formats, and vocabularies, as well as emerging technologies like AI, ML, and NLP, by implementing a query-based or pull-based model to fetch data from various authorized sources. The data may come from the Research centers, PHC, POC, medical institutions *via* electronic health records (EHR), or an open source that is acceptable for analysis after data engineering through contact tracing & tracking to prepare it for processing (Bengio et al., 2020; Dar et al., 2020; Ferretti et al., 2020; Kretzschmar et al., 2020; Piotto et al., 2021).

The proposed DMA includes a user workspace for storing, managing, computing, and sharing data and analytic findings with collaborators or the greater research community. Furthermore, the architecture provides a platform for users and researchers to contribute ideas or proposals that may be implemented by adhering to the DMA's correct principles and policies. It enables data to be pooled between users and researchers and other publicly accessible data *via* the DMA. DMA also provides open data Application Programming Interface (API) to researchers, making data accessible, comprehensible, and actionable, suited to the specific needs of authorized users or qualified researchers.

**Figure 4:**
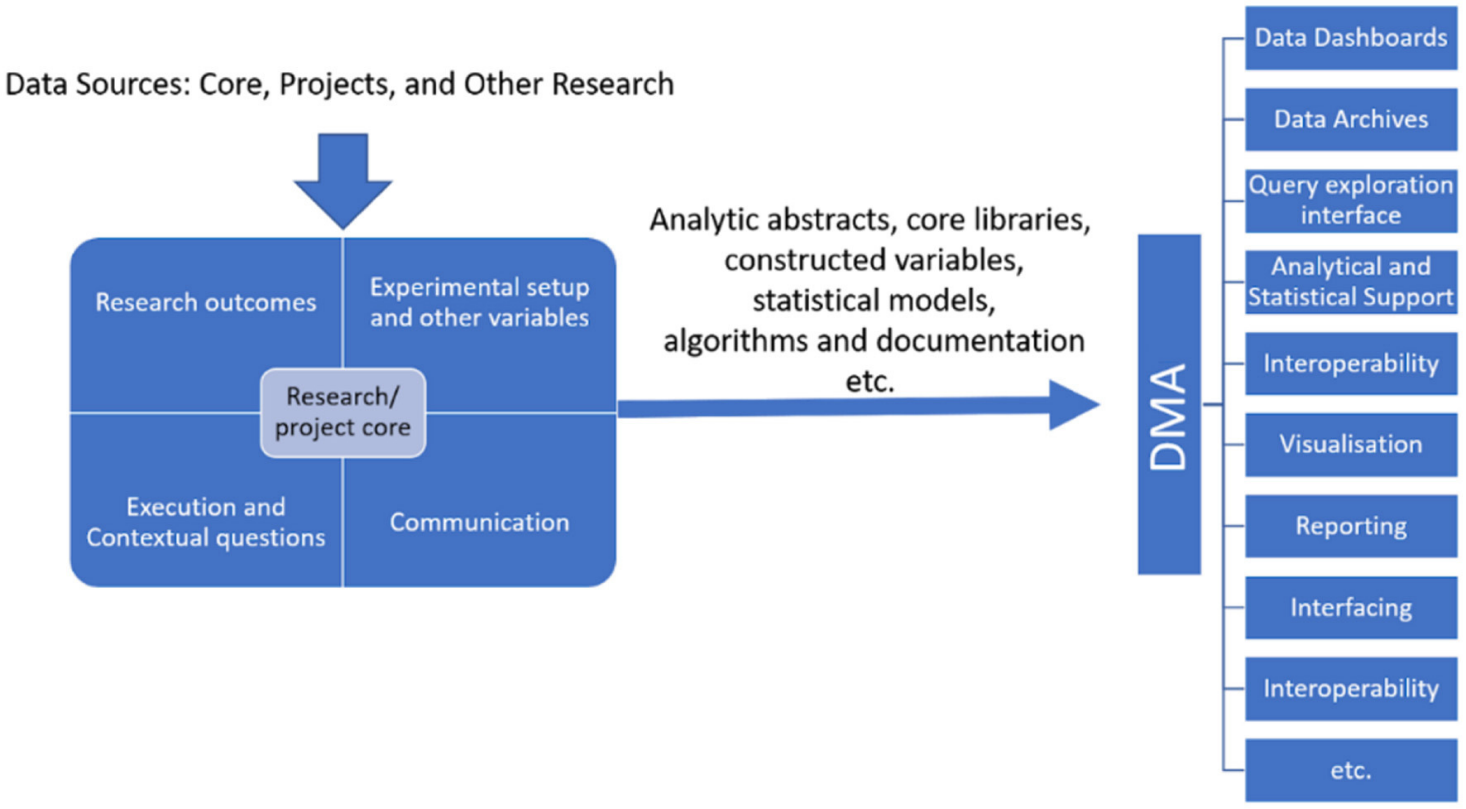
Dataflow across stakeholders in UAE and India.

The DMA enables portable (including wearable) technology deployment. The tools and technologies will be shared with peer researchers/research labs or given free to willing participants. Additionally, open-data APIs will be built to share with diverse collaborators *via* responsive/progressive software applications (compatible with Web/Mobile). The proposed DMA work is open-source and may be accessible using GIT, GITHUB, or other commercial platforms. By providing a web interface and API access to data, tools, and computation and will be made compatible for interaction with other systems. DMA idea serves a wide variety of users, including computer illiterate and computationally adept users. This is accomplished through the availability of resources in the proposed architecture that comprises the web portal through which users can have secure access to the data/application. The web application would also include user manuals and a few sample demonstrations to help beginners and expert users become acquainted with the developed applications. Figure 4 displays the data flow among many stakeholders to achieve a successful DMA.

#### Data Analysis

The DMA concept provides practical and strategic leadership for managing health science innovations in India and UAE. The DMA would work independently and synergistically to meet the purpose and identify the selected focus areas and strategic initiatives to create a rapid, reliable, and scalable research and innovation system. The specific activities mentioned below aim to achieve the DMA concept over 4–5 years.

**Figure 5:**
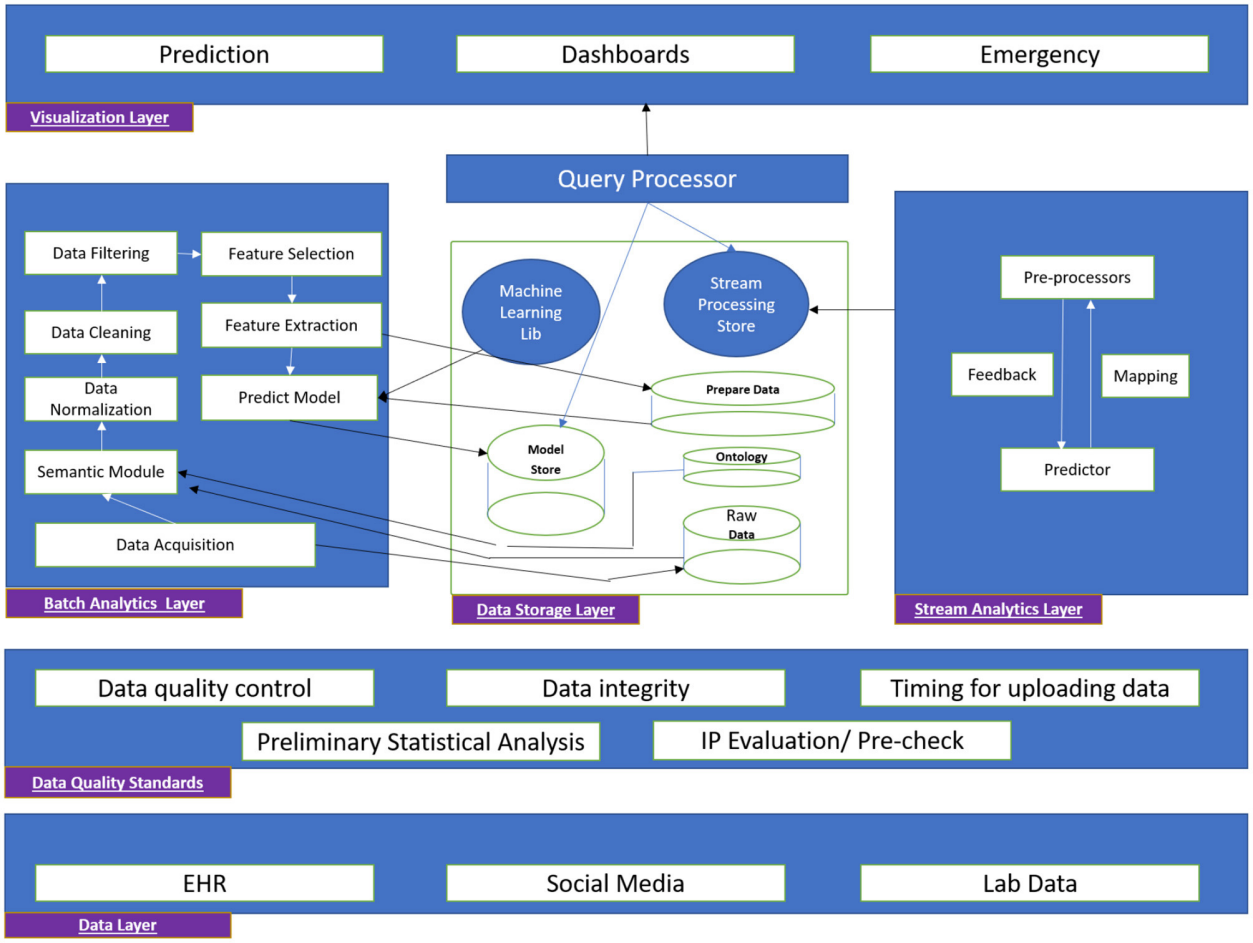
Flow and Interaction of DMA Activities for research concept Goal 2 & Goal 3.

The flow and interaction of the above activities are summarized in Figure 5.

- Deliver a software solution that would improve discoverability. A proposal request would be sent to all the consortium research centers, PHC, POC, and health practitioners, requesting an interface to access the data. Thereby, we can access the data by posing queries or processing the events generated by the data sources, such as Enterprise Data, 3rd Party Cloud Data, social media, etc., if there is an update in the data source. Anaconda tool can be used here.
- Assist the collaborating stakeholders as a technical resource for applicable use cases, including system capabilities to meet international data protection and anonymization regulations. This is accomplished by providing:
  - Support for data volume, variety, and computational requirements.
  - Measuring and Sharing metrics for performance and consumption.
- Encourage industry cooperation to harness current technology and solutions that are both cost-effective and sustainable through:
  - Creating a sustainable and transparent service cost model
  - Serving a variety of users.
  - Adherence to authentication and policies across the globe
  - Adherence to the data anonymization and data protection protocols while maintaining interoperability with other healthcare components.

## Policy Compliance Issues

### Data Standards

The development and deployment of digital health-related facilities are being regulated by National Digital Health Mission (NDHM). Additionally, the federal national health information architecture enforced the integration of information systems related to public and private health. For integrating systems, EHR with consistent Metadata and Data Standards (MDDS) would be supported by this policy. Affordable and innovative ICT would integrate health information into comprehensive EHR. The data quality and standards layer in Figure 5 depicts the data quality standards applied for DMA.

The EHR would be standards-compliant, cost compliant, and enable effective and seamless data transfer between stakeholders. The typical HER data can be of one of the categories related to audio, video, X Rays, Images, etc. These data can be compressed and shared from PHC to healthcare professionals for diagnosis/further actions. ICT-based interventions like reminders using SMS/WhatsApp or other preferred social media apps for patient treatment compliance, recording symptoms, and identifying emergencies would be performed. All medical transactions occurring in the system would be recorded as immutable transactions on a blockchain. EHR-based privacy preserved datasets would be generated and published to study diseases. Analyzing these datasets would aid in public health policy design and implementation. Evidence-informed policymaking (EIPM - WHO, 2017) is considered a critical building block for enhancing the population's health in low, middle, and high-income regions/countries worldwide.

### Ethical Issues (E), Legal Issues (L), Cultural and Social Implication Issues (CS) Would Be Handled in Data Collection, Data Usage and Result Dissemination to Stakeholders Handled Would Be Handled as Detailed Below

The concept is a healthcare concept, and patient data will be collected. Ethical issues concerned with this concept will be taken care of as given below:

- The consent form is prepared. The patient/user who participates in this concept will be informed about the details of the collected data, and the consent form's signature is taken. (E, L)
- The concept does not include clinical intervention because there is no need for ethical clearance. (E)
- For data collection from the ERP of the hospitals, a mutual agreement is required to be signed indicating what type of data is shared for this concept. (L)
- The collected data, if not in digital form, is converted to digital form. The data values like patient name, gender, religion etc., are watermarked/ hidden/removed to avoid personal details of a person/patient. This takes care of patient privacy protection. (E, L, CS)
- The data analysis from collected data will be published as a population study, not highlighting any person in particular. (CS, E)
- The data will be stored in a particular designated system to which the access will be password protected. The cloud storage will be under a specific collaborative organization's name. (L)
- Documentation related to the above is regularly maintained. (E, L)
- The regular periodical meeting will be conducted either online/or offline, and emails will be used to disseminate the results to the stakeholders.

Although the project is focusing on the technical and implementation issues from big data platform and solutions perspectives, we cannot overlook the issues created by its possible global implementations such as ethics, policies, and legal issues that face computer professionals and data scientists while working with health and personal datasets, as well as the project, will examine the related cyber security issues. Further, the ethical issues such as defining ownership of data, obtaining consent to collect and share data, protecting the identity of human subjects and their personal identifying information, and the licensing of data.

There are generally four matters of data acquisition and management that need to be addressed at the outset of a study: (1) collection, (2) storage; (3) ownership, and 4) sharing. These cases and role play present common scenarios that occur at various stages of data acquisition and management. It also includes acquiring sensitive data, sharing data with colleagues, and managing data collection processes.

### Data Management and Resource Sharing Plan

Tools, materials, and protocols from the research activities will be distributed to peers and interested research groups. The dissemination of research findings will be made public with suitable agreements. Based on the guidelines of the Indian and UAE governments, the policies related to sharing the data and conceptual information will be carried out. The research findings will be published in peer-reviewed journals as research manuscripts or through poster presentations or workshops will be conducted for all the interested research individuals/groups to disseminate the information.

### Confidentiality

The UD, NMIT, MBRSM, and RMCH Offices of Research & Innovation (ORI) will manage all knowledge transfer sessions for resources generated by the researcher of all partner institutions and their collaborators. The ORI shall adhere to all relevant policies, principles, guidelines, and procurement regulations in making unique research resources freely available for research purposes to eligible persons and businesses. The ORI will assess and submit for patent protection on any subject innovation that may result from the research concept. Unpublished data that is considered proprietary or secret must be disseminated in accordance with a proper confidential disclosure agreement approved by the ORI. Patient data must be de-identified and disseminated according to ORI rules and relevant laws and regulations. Through an explicit “Software Sharing Plan,” material transfer agreements and other licensing agreements will be formed to share resources across the academic community for non-commercial research usage (SSP).

### Software Sharing Plan

The current proposal would explicitly commit to the following:

- The software from the research concept will be accessible under an open license for research and academic purposes. The tools and findings from the research will be shared with biomedical researchers, research institutions, and the govt. Research laboratories.
- The terms of would allow for the circulation and sale of enhanced or updated versions of the software and the addition of the program into other software packages.
- To maintain community utility, the program would be transferrable so that other individuals or teams might continue development if the original investigators were unable or unable to do so.
- Software availability parameters would include researchers' access to update the source code and share it with other peers. To increase the potential impact of the software developed in this proposal, the proposal would create a plan to manage and disseminate the improvements or customizations of their tools and resources by others using an open revision control and source code management system like GitHub to foster a community of code contributors and allow transfer to another individual/team. This strategy would also specify the conditions of the software availability enabling reuse, modification, and monetization to continue development.

## Innovation

The first innovation in this research concept is defining and using a globally unique identifier (GUI) as a link between the objects containing clinical information and users. This ensures integrity and consistency (anti-tampering systems, anti-data tampering) between the information associated with users and, at the same time, ensures the anonymity of the same. The second innovation is about the extreme level of abstraction chosen to represent the GUI that allows direct mapping of the relationships between the objects in a format navigable by automated data mining and data analysis systems in DMA through the development of algorithms that are more accurate in identifying correlations between “clinical examinations” and “clinical diagnoses”. These relationships would allow expressing the epidemic trend from the point of view of the clinical situation rather than from the epidemiological point of view (which is currently the trend). The third innovation is designing a framework that will provide the essential functions of contact tracing and anonymous GPS tracking through a VHC App. The design framework will focus on the vulnerability analysis of the possible sensor systems that can be used to provide a system as secure as possible within the limits of current technologies.

## Limitation and Challenges

As a limitation, the proposed conceptual model might encounter deployment challenges and ease of adoption by the stakeholders and therefore requires hand-holding with all stakeholders for smooth and quicker implementation. The challenges and limitations are n dimensional. Dimensions can be increasing as the implementation of the proposed conceptual model start to progress.

On the broader note the major challenges of realizing the proposed conceptual model lies in the following points

- The disparate healthcare landscape and operational silos
- Rapid changes in healthcare data sources, types, volumes
- Positioning EMR/EHRs to absorb the increasing changes in data
- Stringent industry regulations on how data is handled
- Unique challenges faced by providers, payers, patients
- Need for more maturity in clinical and non-clinical data capture mechanisms

Few other limitations of realizing the proposed conceptual model can be Regulations and Compliance, Solving Regulatory Concerns, Volume Issues, Fragmented Data etc.

The proposed conceptual framework is applicable in multiple contexts and can quickly scale up to millions of users in multiple countries at an affordable cost. Heterogeneous devices leave the computational load to the distributed network. This will lead to countless possibilities for developing new solutions and new integrations with existing systems. The open-access and third-party applications that can be marketed and with a high market value will be, in addition to those already mentioned in the concept, will result in considerable savings for the public health system in multiple countries globally.

## Conclusion

The proposed research concept aims at augmenting the PHC and POC system with ICT-based interventions to enable cost-effective healthcare delivery and potentially change the health outcome and result in better health for patients. The conceptual design is open, flexible, and scalable, and it allows users to access a wide range of digital materials. The data format is also extendable to access additional data/metadata, and the architecture is scalable to accommodate petabyte-scale federated discovery. Existing technology and resources are used to implement modular DMA. The uniqueness of the concept lies in the fact that the conceptual framework provides patients, including their data, access to specialist doctors from around the globe *via* the VHC; Improved health due to compliance to holistic disease treatment plans, and access to scientific health information; and Reduction in OoPE to patients.

## Research Milestones (Work Plan - Planned Activities With Gant Chart)

The concept will be led by Dr. Manoj Kumar (Department of Information Science and Engineering) with support from his NMIT colleagues viz., Dr. Jagadish Patil, Dr. K Aditya Shastry, Dr. Shiva Darshan (Department of Computer Science and Engineering); Dr. Nanda Kumar - from RMCH in India; in addition to Dr. Immanuel Azaad Moonesar from MBRSG-Dubai, and Dr. Nasser Al Muraqab from UD in Dubai. Prof. Ananth Rao from UD-Dubai, with the consortium members' assistance, will monitor and evaluate the concept work plan and outcomes for timely completion of the concept with financial assistance from MOH, DHA, and ADHA in UAE, and ICMR-New Delhi-India, besides the health industry partners. The plan of action and research milestones are detailed in Table 1.

**Table 1:** Plan of action and research milestones.

**Activity**	**Year 1**				**Year 2**				**Year 3**				**Year 4**				**Year 5**			
	**Q1**	**Q2**	**Q3**	**Q4**	**Q1**	**Q2**	**Q3**	**Q4**	**Q1**	**Q2**	**Q3**	**Q4**	**Q1**	**Q2**	**Q3**	**Q4**	**Q1**	**Q2**	**Q3**	**Q4**
Formulation of functional requirements, VHC and DMA architecture, and design decisions																				
Documentation, dissemination of information among the stakeholders																				
Designing the plan and setting up the infrastructure																				
Discovery of data sources and negotiation involving policies and rules to be incorporated for data sharing																				
Raw data storage																				
Data Engineering																				
Metadata management & Warehouse identification																				
Data classification																				
Multiple algorithm creation and Data Modeling																				
Data Analytics and Research problem identification Exploration of open data APIs																				
Assembling all the modules into a unified model (VHC and DMA)																				
Designing of VHC and Data Management policies and their implementation																				
Formulation of data visualization and generation of reports																				
Self-Audit trails and Web Platform design																				
Full VHC and DMA deployment and testing, Scalability of VHC and DMA Integration with Business rules, Enterprise governance																				

## Author Contributions

AR laid the foundation and coordinated with the team to bring out the paper's content. His significant contributions are establishing the Background, Research Problem, and Objectives of this conceptual paper. KS and JP worked on Design/Conceptual Framework for Goal 1 and Objectives O.1.1 to O.1.4. SD and NS have worked on Design/Conceptual Framework for Goals 2 & 3 and its related objectives The sections Innovation, Limitation and Challenges, and Conclusions are contributed significantly by IM, NA, and MK has written the manuscript by coordinating with all the authors. He made all the edits, referencing, abstract, conclusion, and communication with the group and brought the contents of the manuscript to the current shape. SA has greatly contributed during the conceptualizing phase, his contributions are greatly in articulating the contents of this manuscript All authors are involved in conceptualizing the paper's contents and discussing the contents/design/flow of the paper. All authors contributed to the article and approved the submitted version.

## Conflict of Interest

The authors declare that the research was conducted in the absence of any commercial or financial relationships that could be construed as a potential conflict of interest.

## Publisher's Note

All claims expressed in this article are solely those of the authors and do not necessarily represent those of their affiliated organizations, or those of the publisher, the editors and the reviewers. Any product that may be evaluated in this article, or claim that may be made by its manufacturer, is not guaranteed or endorsed by the publisher.

## References

[B1] Baig M. M. Gholamhosseini H. Moqeem A. A. Mirza F. Lindén M. (2017). Clinical decision support systems in-hospital care using ubiquitous devices: current issues and challenges. Health Informat. J. 25, 1091–1104. 10.1177/1460458217740722 29148314

[B2] Bengio Y. Janda R. Yu Y. W. Ippolito D. Jarvie M. Pilat D.. (2020). The need for privacy with public digital contact tracing during the COVID-19 pandemic. Lancet Dig. Health 2, e342–e344. 10.1016/S2589-7500(20)30133-3 32835192 PMC7266569

[B3] Dar A. B. Lone A. H. Zahoor S. Khan A. A. Naaz R.. (2020). Applicability of mobile contact tracing in fighting pandemic (COVID-19): Issues, challenges, and solutions. Comp. Sci. Rev. 38, 100307. 10.1016/j.cosrev.2020.100307 32989380 PMC7510441

[B4] EIPM - WHO (2017). Evidence-Informed Policy Making (EIPM) is the Process of Using Research Evidence in Health Policy to Strengthen Health Systems to Benefit the Health of the Wider Population. World Health Organization. Available online at: https://www.gavi.org/our-alliance/global-health-development/sustainable-development-goals (accessed March 7, 2022)

[B5] Ferretti L. Wymant C. Kendall M. Zhao L. Nurtay A. Abeler-Dörner L.. (2020). Quantifying SARS-CoV-2 transmission suggests epidemic control with digital contact tracing. Science 368, eabb6936. 10.1126/science.abb6936 32234805 PMC7164555

[B6] Gadekallu T. R. Khare N. Bhattacharya S. Singh S. Maddikunta P. K. R. Srivastava G. (2020). Deep neural networks to predict diabetic retinopathy. J. Ambient Intell. Humaniz. Comput. 10.1007/s12652-020-01963-7

[B7] Gomez E. A. Katia P. (2010). Information and communication technologies (ICT) options for local and global communities in health-related crisis management. J Commun Informat. 6, 2.

[B8] HDI (2020). Under United Nations Development Program (UNDP) 2020 report-Human Development Index (HDI).

[B9] Healthconnect-intl.org (2020). ICT For Health — Healthconnect International. Available online at: http://www.healthconnect-intl.org/ictforh.html (accessed March 24, 2020).

[B10] Kabelac Z. Tarolli C. G. Snyder C. Feldman B. Glidden A. Hsu C.-Y.. (2019). Passive monitoring at home: a pilot study in Parkinson's disease. Digital Biomark. 3, 22–30. 10.1159/000498922 32095766 PMC7015389

[B11] Kretzschmar M. E. Rozhnova G. Bootsma M. C. van Boven M. van de Wijgert J. H.. (2020). Impact of delays on the effectiveness of contact tracing strategies for COVID-19: a modeling study. Lancet Public Health 5, e452–e459. 10.1016/S2468-2667(20)30157-2 32682487 PMC7365652

[B12] Li J. Moore N. Akter S. Bleisten S. Ray P. (2010). mHealth for influenza pandemic surveillance in developing countries, in 43rd Hawaii International Conference on System Sciences (Kauai, Hawaii, USA).

[B13] Li J. Ray P. Seale H. Macintyre R. (2012). An E-health readiness assessment framework for public health services–pandemic perspective, in 2012 45th Hawaii International Conference on System Sciences (Maui, Hawaii USA).

[B14] National Academy of Sciences (2015). The Role of Telehealth in an Evolving Health Care Environment -Workshop Summary. Washington, DC: The National Academies. Available online at: http://www.iom.edu/en/Reports/2012/The-Role-of-Telehealth-in-an-EvolvingHealth-Care-Environment (accessed January 1, 2022)

[B15] Numan M. Subhan F. Khan W. Z. Hakak S. Haider S. Reddy G. T.. (2020). A systematic review on clone node detection in static wireless sensor networks. IEEE Access 8, 65450–65461. 10.1109/ACCESS.2020.2983091

[B16] PAHO (2011). Strategy and Plan of Action on eHealth. Washington, DC: Pan American Health Organization. Available online at: https://iris.paho.org/bitstream/handle/10665.2/28414/9789275119037_eng.pdf (accessed February 3, 2022)

[B17] Piotto S. Di Biasi L. Marrafino F. (2021). Evaluation of epidemiological risk using contact tracing open data. J. Med. Internet Res. 23. 10.2196/preprints.28947 PMC8330631 34227997

[B18] Sandhu R. Gill H. K. Sood S. K. (2016). Smart monitoring and controlling of Pandemic Influenza A (H1N1) using Social Network Analysis and cloud computing. J. Comput. Sci. 12, 11–22. 10.1016/j.jocs.2015.11.001 32362959 PMC7185782

[B19] Scott R. E. (2009). Global e-health policy–from concept to strategy, in Telehealth in the Developing World, eds Wootton R. Patel N. Scott R. E. Ho K. (London: Royal Society of Medicine Press), 55.

[B20] Shaikh A. T. Ferland L. Hood-Cree R. Shaffer L. Mcnabb S. J. N. (2015). Disruptive innovation can prevent the next pandemic. Front. Public Health 3, 215. 10.3389/fpubh.2015.00215 26442242 PMC4585064

[B21] Yu K.-H. Beam A. L. Kohane I. S. (2018). Artificial intelligence in healthcare. Nat. Biomed. Eng. 2, 719–731. 10.1038/s41551-018-0305-z 31015651

[B22] Zhu H. Podesva P. Liu X. Zhang H. Teply T. Xu Y.. (2019). IoT PCR for pandemic disease detection and its spread monitoring. SSRN Electron. J. 303. 10.2139/ssrn.3352008 32288256 PMC7125887

